# Clinical hypertension: a new era

**DOI:** 10.1186/2056-5909-20-7

**Published:** 2014-09-25

**Authors:** Jong-Won Ha

**Affiliations:** Yonsei University Severance Cardiovascular Hospital, Seoul, South Korea

## Abstract

We are delighted to announce the launch of the new journal *Clinical Hypertension. Clinical Hypertension* is an open-access, peer-reviewed, online journal, which will publish scientific investigation of the highest quality in the field of blood pressure regulation and pathophysiology, treatment, and prevention of hypertension. The editors encourage the submission of original articles that deal with basic, clinical, and population studies of hypertension and related fields, such as cardiology, nephrology, endocrinology, neuroscience, vascular biology, physiology, pharmacology, cellular and molecular biology, and genetics.

## Editorial

*Clinical Hypertension* is a continuation of the *Journal of the Korean Society of Hypertension* which has now transferred to the open-access publisher BioMed Central. As an open-access journal, anybody with access to the Internet will be able to freely access and download all published materials in *Clinical Hypertension*. It will therefore provide unrestricted access to new scientific data for clinicians and scientists. Open-access publishing is supported by an article-processing charge, paid by the authors or their supporting institution, or funding body. For *Clinical Hypertension*, this charge is covered by the Korean Society of Hypertension, meaning that it is effectively free to publish for authors.

The Korean Society of Hypertension was founded in 1994 in order to provide a meeting for all scientists with an interest in hypertension. Since that time, the Society has organized a number of annual academic as well as educational meetings and is now one of the biggest academic meetings in Korea, with over 2,887 members. The society includes six working groups, covering varying aspects of hypertension, such as epidemiology, pediatric hypertension, blood pressure monitoring, and target organ damage. They also work on a number of public health campaigns in order to raise awareness and increase prevention of hypertension-based diseases.

Hypertension-related diseases are the greatest contributor to the burden of cardiovascular diseases and death worldwide, and it is a global problem of major significance. *Clinical Hypertension* therefore aims to support the community by making new research in the field as widely and openly available as possible.

Although *Clinical Hypertension* is the official journal of the Korean Society of Hypertension, we would like to encourage colleagues from across the world to consider our journal for their publications. This internationalization contributes to the motivation behind our move to BioMed Central. As an online journal, *Clinical Hypertension* is widely available to colleagues across the globe. We believe that this international approach to publishing research is important, as the sharing of data worldwide allows greater development to both research studies and clinical applications.

This launch has been made possible by a large number of people, and in particular, I would like to thank the Board of the Korean Society of Hypertension and the members of the journal editorial board, who have given their full support.

With the launch of *Clinical Hypertension*, we are entering a new era of publishing in the field of hypertension and vascular diseases. On behalf of the entire editorial board, I would like to welcome you to this exciting new journal.

